# Time from injury to acute surgery for patients with traumatic cervical spinal cord injury in South-East Norway

**DOI:** 10.3389/fneur.2024.1420530

**Published:** 2024-06-24

**Authors:** Mads Aarhus, Jalal Mirzamohammadi, Pål Andre Rønning, Mona Strøm, Thomas Glott, Syed Ali Mujtaba Rizvi, Donata Biernat, Håvard Ølstørn, Pål Nicolay Fougner Rydning, Vidar Tveit Vasfaret Stenset, Pål Aksel Næss, Christine Gaarder, Tor Brommeland, Hege Linnerud, Eirik Helseth

**Affiliations:** ^1^Department of Neurosurgery, Oslo University Hospital, Oslo, Norway; ^2^Institute of Clinical Medicine, Faculty of Medicine, University of Oslo, Oslo, Norway; ^3^Spinal Unit, Sunnaas Rehabilitation Hospital, Nesodden, Norway; ^4^Department of Neuroradiology, Oslo University Hospital, Oslo, Norway; ^5^Department of Traumatology, Oslo University Hospital, Oslo, Norway

**Keywords:** cervical spinal cord injury, timing of surgery, patient transfer, improvement of care, neurotrauma

## Abstract

**Background:**

The recommended treatment for cervical spinal cord injury (cSCI) is surgical decompression and stabilization within 24 h after injury. The aims of the study were to estimate our institutional compliance with this recommendation and identify potential factors associated with surgical delay.

**Methods:**

Population-based retrospective database study of patients operated for cSCI in 2015–2022 within the South-East Norway Health Region (3.1 million inhabitants). Data extracted were demographics, injury description, management timeline, place of primary triage [local hospital (LH) or neurotrauma center (NTC)]. Main outcome variables were: (1) time from injury to surgery at NTC, (2) time from injury to admission NTC, and (3) time from admission NTC to surgery.

**Results:**

We found 243 cSCI patients having acute neck surgery. Their median age was 63 years (IQR 47–74 years), 77% were male, 48% were ≥65 years old. Primary triage at an LH occurred in 150/243 (62%). The median time from injury to acute surgery was 27.8 h (IQR 15.4–61.9 h), and 47% had surgery within 24 h. The median time from injury to NTC admission was 5.6 h (IQR 1.9–19.4 h), and 67% of the patients were admitted to the NTC within 12 h. Significant factors associated with increased time from injury to NTC admission were transfer via LH, severe preinjury comorbidities, less severe cSCI, time of injury other than night, absence of multiple injuries. The median time from NTC admission to surgery was 16.7 h (IQR 9.5–31.0 h), and 70% had surgery within 24 h. Significant factors associated with increased time from NTC admission to surgery were increasing age and non-translational injury morphology.

**Conclusion:**

Less than half of the patients with cSCI were operated on within the recommended 24 h time frame after injury. To increase the fraction of early surgery, we suggest the following: (1) patients with clinical suspicion of cSCI should be transported directly to the NTC from the scene of the accident, (2) MRI should be performed only at the NTC, (3) at the NTC, surgery should commence on the same calendar day as arrival or as the first operation the following day.

## 1 Introduction

Cervical spinal cord injury (cSCI) is a possible devastating complication after trauma to the head and neck region. The optimal patient outcome depends on acute care and rehabilitation. Early surgical decompression of the spinal cord is regarded as one of the key factors to potentially limit spinal cord injury and increase the likelihood for neurological improvement. Various time frames have been proposed to define early surgical intervention post-injury (1–5). The 24 h threshold has been widely adopted as a target for initiating surgical interventions following trauma. Recent guidelines have reinforced this time frame, recommending it to achieve optimal patient management and outcomes (6).

In Norway, all surgical intervention for cSCI is performed by neurosurgeons at the regional university hospitals where the neurotrauma centers (NTC) are located. Some patients with acute cSCI are transported directly from the scene of accident to the NTC, while most patients are first triaged at local hospitals (LH) before transfer to the NTC. Complying with the recommended 24 h threshold at the NTC requires that the LH ensure swift “door-in-door-out” times for interhospital transfers. This necessitates an efficient transfer system to facilitate rapid patient movement. Additionally, the NTC must minimize the duration from patient arrival (“door-in”) to the commencement of surgery.

Numerous countries have structured their health-care systems to meet the standards necessary for timely acute surgical care, particularly for patients with traumatic cSCI. However, there is a scarcity of population-based research assessing whether these health-care systems ensure that such patients receive surgery within the critical 24 h window post-injury. One particular issue is whether patients with cSCI should be directly transferred to the NTC so early surgery can be provided. This has been reflected in a recent North American report of 724 patients with spinal cord injury, where 40% of the patients were first triaged at an LH (7). Furthermore, there is a lack of studies specifically addressing door-in to surgery start time at the NTCs. Such studies can reveal potential targets for improvement for the fraction of patients needing surgery within 24 h of injury.

Here, we present population-based, real world, contemporary data of the time line from injury to acute cervical surgery for patients with traumatic cSCI in a health-care system with a well-organized interhospital transfer system, and a 24/7 service for acute surgery at the regional NTC. Within the Norwegian health-care system, we hypothesize, it would be reasonable to expect the following for patients with traumatic cSCI: (1) that >75% of patients be admitted to the NTC within 12 h of injury; (2) that >75% of patients undergo surgery within 24 h of injury. In line with these hypotheses, the aim of our study was to explore the extent to which these time-line goals have been met and to possibly identify factors that can be further improved to reach the goals.

## 2 Materials and methods

Oslo University Hospital (OUH) is a major Scandinavian trauma center covering the South-East Norway Health Region (SENHR) with a catchment area of 3.1 million people. OUH serves as the NTC and sole provider of trauma-related cervical surgeries for this population. There are 20 LHs within the SENHR with general and/or orthopedic surgeons and radiological services that refer patients with head and cervical spine injuries to OUH. Most patients with cSCI are admitted to OUH for acute phase management, with the exception of a few patients handled conservatively at the local hospitals after consultation with the NTC neurosurgeons on call. In Norway, all neck surgery is performed by neurosurgeons.

Acute management of patients with cSCI at OUH follows standard recommendations (8–14). In brief, patients are treated in the neurointensive care unit (NICU) with continuous monitoring of vital functions, elevation of the mean arterial blood pressure (MAP) > 85 mmHg for 5–7 days, ventilation support if needed, thromboprophylaxis with stockings and low molecular weight heparin (LMWH), pressure wound prophylaxis, enteral nutrition, elimination surveillance, and early rehabilitation, as well as management of multiple injuries. In line with recent evidence, we do not treat cSCI patients with methylprednisolone (15, 16). All patients with neck trauma and neurological deficits are evaluated with MRI before surgery. Acute surgical decompression/stabilization is recommended within 24 h of injury in patients with indication for surgery and who are deemed fit to tolerate the operative procedure. We have state-of-the-art operating rooms (ORs) available 24/7 with qualified neurosurgeons, OR nurses, and anesthesiology teams. Standard intraoperative fluoroscopy is used for all procedures. Neuronavigation based on preoperative CT is used for screw placement in C1 and C2 and for pedicle screws in C6–Th2, while lateral mass screws are mainly placed freehand (navigation is used if considered necessary). The surgical procedures that can be performed 24/7 are anterior cervical decompression and fusion (ACDF) with plating, corpectomy with bone graft and plating, odontoid screws, Harms fixation, posterior screw fixation with rods ± laminectomy, and laminectomy alone. In the study period, patients were routinely prescribed a Miami collar for 6 weeks postoperatively. Patients with persistent neurological deficits are referred to rehabilitation.

Since 1 January 2015, the Department of Neurosurgery at OUH has registered all consecutive cases of cervical spine fractures (CS-Fx) from C0/1 to C7/Th1 with or without concomitant cSCI that are referred to the department in a dedicated database. Only patients with a Norwegian social security number and living within the SENHR are included in the registry. Included are all cervical fractures, discoligamentous complex (DLC) injuries causing cSCI or in need of stabilization (external immobilization or surgery), and all traumatic cSCIs (17). The following cSCIs are not included: injuries secondary to spontaneous cerebrovascular catastrophes, spinal tumors, myelopathy secondary to degenerative cervical spine stenosis without trauma, and cSCIs as a complication to surgery.

**Figure 1:**
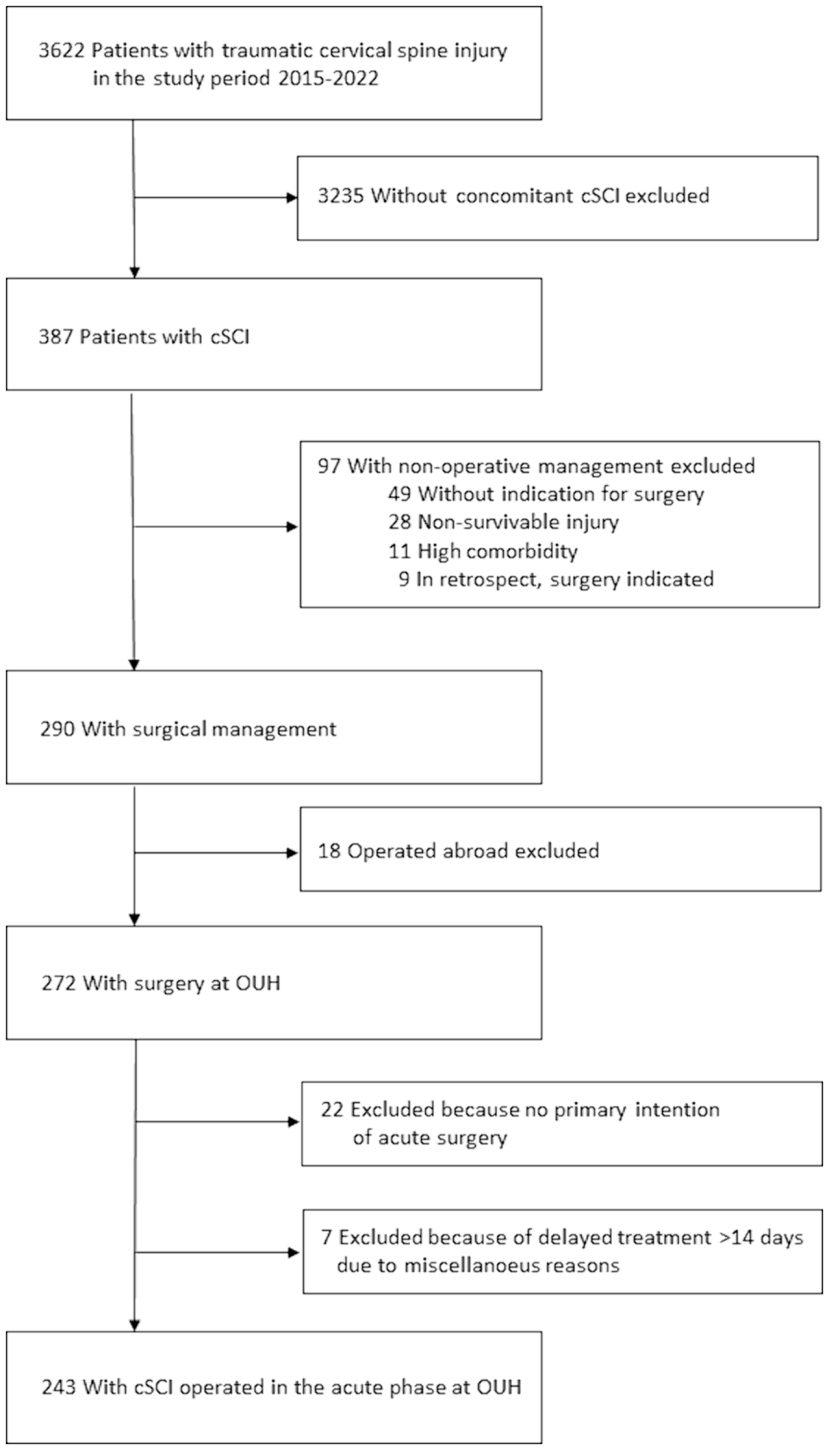
Overview of the patient population and exclusion criteria.

For the study, we investigated only patients undergoing surgical management of cSCI in the acute phase at our own institution. Thus, from the database, we excluded cases of traumatic cervical spine injuries without concomitant cSCI (*n* = 3,235), cases that were non-operatively managed (*n* = 97), cases operated at other institutions (*n* = 18), cases without primary intention of surgery (*n* = 22), cases with primary intention of surgery who had the procedure delayed more than 14 days after injury for various reasons (*n* = 7; Figure 1).

From the database, we retrieved the following data: date and time of injury, date and time of admission to OUH, admitted to OUH from scene of accident or local hospital, sex, age at time of injury, living status at time of injury (home—care for self, home—but need assistance with activities of daily life (ADL), or institutionalized), preinjury-ASA score (American Society of Anesthesiologists Physical Status Classification system) (18) (1: normal healthy; 2: mild systemic disease; 3: severe systemic disease; 4: life threatening systemic disease), ankylosing spondylitis (no or yes), diffuse idiopathic skeletal hyperostosis (DISH; no or yes), preinjury cervical spinal stenosis (no or yes), mechanism of injury (categorized as fall, bicycle (including e-bike and e-scooter), four-wheel motor vehicle accident (4-W MVA), two-wheel motor vehicle accident (2-W MVA), skiing, diving, other sports/play/recreation activities, and other), anatomical level of cSCI, cSCI classified according to the American Spinal Injury Association Impairment Scale (AIS) (19) into grade A (complete), B-C-D (incomplete), E (none), type of cSCI (central cord syndrome (CSS) or non-CCS), morphological classification of the cervical spine injury, multiple trauma (no or yes). Concomitant head injury was scored according to head injury severity score (HISS) (20) into mild–moderate–severe, cervical imaging (cervical-CT, cervical MRI), date and time of first acute neck surgery, surgical approach (anterior, posterior, 360°), and use of CT based perioperative neuronavigation (no or yes).

Subaxial injuries causing cSCI were classified according to the AO Spine Subaxial Cervical Spine Injury Classification System (21). Injuries to the upper cervical spine (C0–C2) were categorized as odontoid fractures, Hangman fractures, or major DLC injury with distraction/dislocation in the C0-C2 region.

CCS was defined as a cSCI resulting in more pronounced paresis in the arms than legs (22).

Multiple injuries were defined as a simultaneous traumatic brain injury (mild, moderate, or severe, according to HISS) and/or imaging-proven (X-ray, CT, or ultrasound) injury in one or more of the following regions: face, thoracolumbar spine, chest, abdomen, pelvis, or extremities. Skin injuries were not registered.

The main outcome variables were time from injury to surgery, time from injury to NTC admission, and time from NTC admission to surgery.

In our setting, where acute surgery is performed in one single NTC covering a large geographic area with 20 acute care LHs referring patients to the NTC, we found it meaningful to divide the time to surgery into different periods: (1) time from injury to NTC admission and (2) time from NTC admission to surgery. Both time periods influence the time from injury to surgery but may be associated with different time-delaying factors.

Data were summarized using frequencies for categorical data and median values for continuous data. Continuous and categorical variables were compared with the Wilcoxon rank-sum, Kruskal Wallis, and chi-squared tests. To address the right skewness in the distribution of the dependent time variable, we applied a logarithmic transformation. This transformation normalized the data, thereby improving the validity of subsequent statistical analyses and their assumptions. To investigate the effect of different covariates on the transformed time to surgery, we used uni- and multivariate linear regression analyses and assessed assumptions graphically afterward.

The study was approved by the OUH Data Protection Officer (DPO approval no. 23/28298). The need to obtain informed consent from patients was waived. The quality control database for traumatic CS-Fx in southeastern Norway is approved by the OUH DPO (DPO approval no. 2014/12304).

## 3 Results

### 3.1 Patient characteristics

From an analysis of the registry, we identified 243 consecutive patients undergoing acute surgery for cSCI at OUH in the eight-year period 2015–2022 (Figure 1). The median age was 63 years (IQR 47–74 years), 77% were males, 48% were ≥65 years (WHO definition of elderly), 39% had severe comorbidities (preinjury ASA scores ≥ 3), 7% had preinjury dependent living, 68% had a fall injury, 44% had multiple trauma, and 6% had moderate to severe TBI. Further patient characteristics are given in Table 1. Initial patient triage occurred at an LH before transfer to NTC in 150/243 (62%), while 93/243 (38%) were transported directly from the scene of accident to the NTC.

**Table 1:** Characteristics of the 243 patients with traumatic cSCI who were operated on acutely at OUH from 2015 to 2022.

		*N* = 243 (100%)
Age	Age ≥ 65 years	117 (48.1%)
Sex	Male	186 (76.5%)
Preinjury severe comorbidities	ASA ≥ 3	94 (38.7%)
Preinjury dependent living	Home with assistance/Institutionalized	18 (7.4%)
Main morphology C0–C2 injury	Odontoid Fx	11 (4.5%)	Hangman Fx	2 (0.8%)	Major DLC injury	1 (0.4%)
Main morphology subaxial injury	A0—Minor, nonstructural fx	91 (37.4%)	A1—Wedge-compression	5 (2.0%)	A2—Split	5 (2.0%)	A3—Incomplete burst	1 (0.4%)	A4—Complete burst	10 (4.1%)	B1—Posterior tension band injury (Chance)	8 (3.3%)	B2—Posterior tension band injury	4 (1.6%)	B3—Anterior tension band injury	17 (7.0%)	C—Translational injury	88 (36.2%)
	Type of cSCI	Non-central cord syndrome (non-CCS)	149 (61.3%)	Central cord syndrome (CCS)	94 (38.7%)
	ASIA Impairment Scale (AIS)	A	42 (17.3%)	B	33 (13.6%)	C	69 (28.4%)	D	99 (40.7%)
	Admittance	Directly from scene of accident	93 (38.3%)	Via local hospital	150 (61.7%)
	Surgical approach	Anterior fixation	131 (53.9%)	Posterior fixation	63(25.9%)	Anterior + posterior (360°) fixation	37(15.2%)	Posterior laminectomy alone	12 (4.9%)
	Intraoperative image guidance	Standard intraoperative fluoroscopy	243 (100%)	Brainlab CT-based navigation	62 (25.5%)
	Mortality	In-hospital mortality OUH	7 (2.9%)
		30-day mortality	14 (5.8%)

### 3.2 Cervical spine injury morphology

Cervical imaging included cervical spine CT in 243/243 (100%) and cervical spine MRI in 238/243 (98%). The morphological classification of the predominant cervical spine injury is listed in Table 1. The most frequent C0–C2 injury was an odontoid fracture with dislocation of the odontoid fragment. Translation was observed in 12/14 C0-C2 injuries. The two most frequent subaxial injuries were, according to the AO Spine subaxial classification system, “Translational injury (subtype C)” and “Minor non-structural injury (subtype A0).” Per definition, patients with traumatic cSCI with pre-injury cervical spinal stenosis as the major morphological finding on imaging were classified as having a “Minor non-structural injury.” Pre-injury ankylosing spondylitis and DISH were seen in 19/243 (8%) and 6/243 (2%) patients, respectively.

### 3.3 Cervical spinal cord injury description

The primary imaging level of spinal cord pathology was C0–C2 in 12/243 (5%) and subaxial in 231/243 (95%) patients. The clinical type of cSCI was classified as CCS in 94/243 (39%) and non-CCS in 149/243 (61%). CCS was significantly more common than non-CCS in patients with preinjury degenerative cervical spinal stenosis (53/94 vs. 48/149, Pearson chi square *p* < 0.001). The cSCI was AIS grade A in 17% of the patients, B in 14%, C in 28%, and D in 41% (Table 1). Thus, 83% had an incomplete, and 17% had a complete cSCI. Patients with CCS were either AIS grade D (70/94 or 74%) or C (24/94 or 26%), while AIS grades in patients with non-CCS were grade A in 42/149 (28%), B in 33/149 (22%), C in 45/149 (30%), and D in 29/149 (20%).

### 3.4 Time from injury to acute surgery

**Figure 2:**
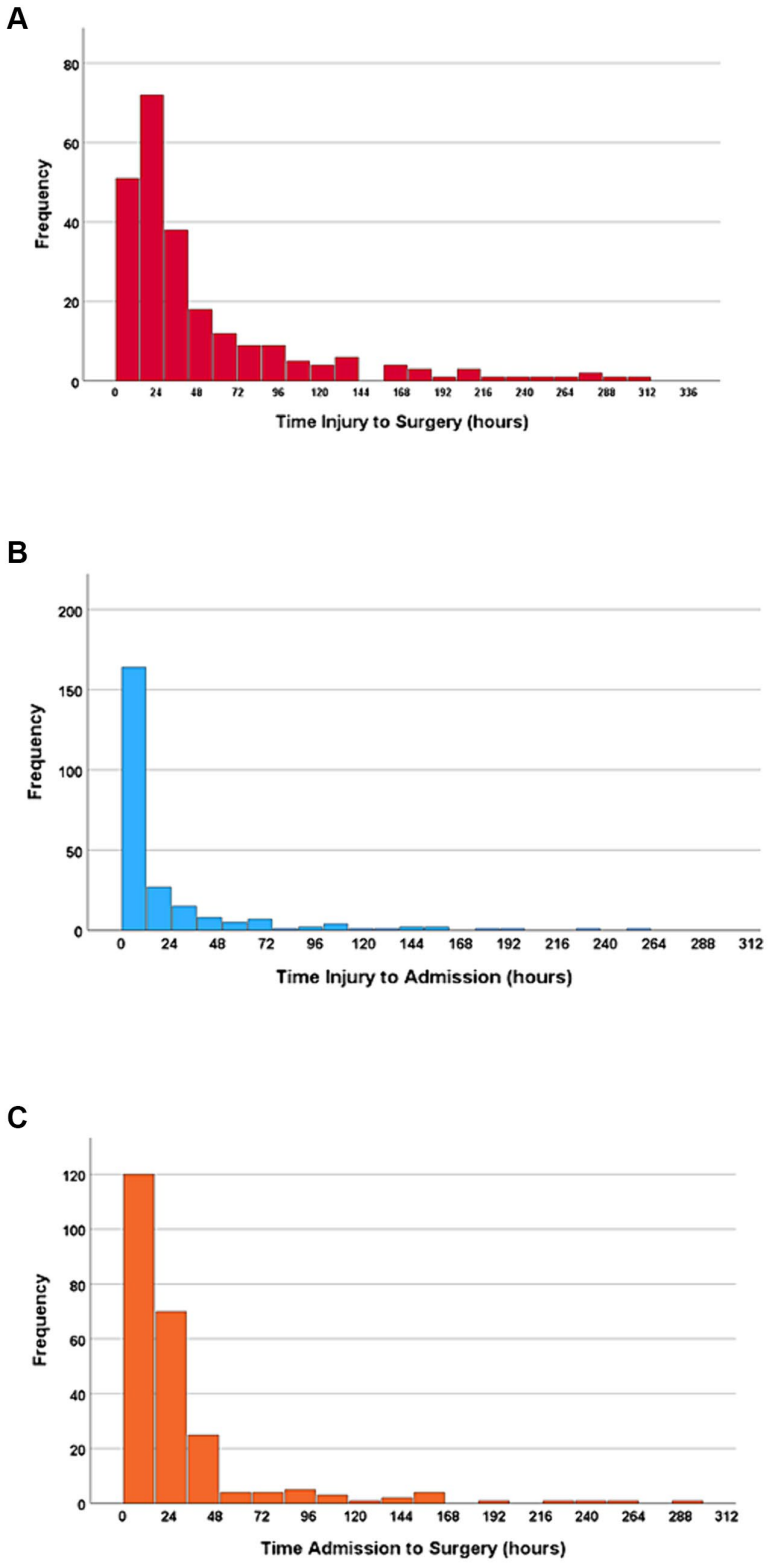
**(A)** Time from injury to surgery. **(B)** Time from injury to NTC admission. **(C)** Time from NTC admission to surgery. *N* = 243.

The median time from injury to acute surgery was 27.8 h (IQR 15.4–61.9 h); 47% had surgery within 24 h, 72% within 48 h, and 79% within 72 h (Figure 2A).

### 3.5 Time from injury to NTC admission

**Figure 3:**
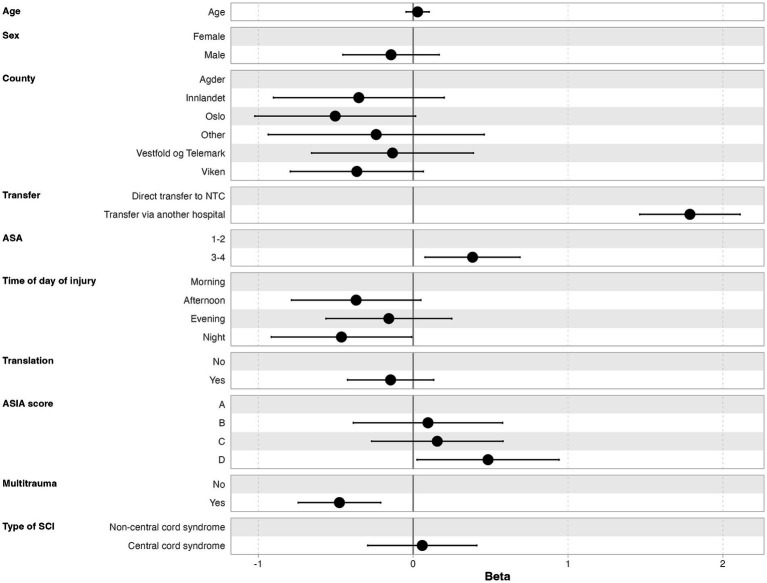
Multivariate linear regression analysis of factors associated with increased time from injury to NTC admission.

The median time from injury to NTC admission was 5.6 h (IQR 1.9–19.4 h), and 67% of the patients were admitted to the NTC within 12 h. Further, 79% of the patients were admitted to the NTC within 24 h after injury, 88% within 48 h, and 92% within 72 h (Figure 2B). In multivariate linear regression analysis, the following factors were significantly associated with increased time from injury to admission at an NTC: transfer via another hospital, severe preinjury comorbidities (ASA ≥ 3), less severe cSCI (AIS = D), time of injury other than night, and absence of multiple injuries (Figure 3).

### 3.6 Time from NTC admission to acute surgery

**Figure 4:**
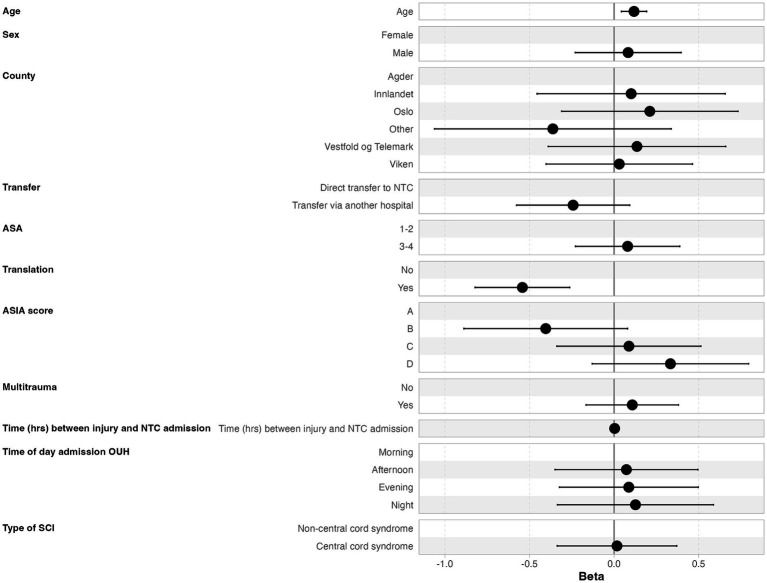
Multivariate linear regression analysis of factors associated with earlier start of surgery after NTC admission.

The median time from NTC admission to surgery was 16.7 h (IQR 9.5–31.0 h), where 70% underwent surgery within 24 h after NTC admission, 89% within 48 h, and 91% within 72 h (Figure 2C). In multivariate linear regression analysis, the following two factors were significantly associated with earlier start of surgery after NTC admission: low age and translational injury morphology (Figure 4).

### 3.7 Surgical treatment

Acute cervical surgeries commenced in the morning for 116 patients (48%), in the afternoon for 88 patients (36%), during the evening for 29 patients (12%), and at night for 10 patients (4%). The surgical approach for the primary acute cervical fixation/decompression was anterior in 131 patients (54%), posterior in 63 (26%), 360° in 37 (15%), and laminectomy alone in 12 (5%). Standard intraoperative fluoroscopy was used in 100%, while CT-based navigation for placement of C1–C2 screws and pedicle screws in C6–Th2 was used in 62/243 (26%) of the procedures.

## 4 Discussion

In this population-based study of patients with traumatic cSCI, 67% of the patients were admitted to the NTC within 12 h after injury, and 47% of the patients were operated on within the recommended 24 h after injury. Thus, our hypotheses that, in our catchment area, >75% of cSCI patients were admitted to the NTC within 12 h after injury and > 75% of the patients were operated on <24 h after injury were rejected. The most significant delay was observed at the NTC, with a median duration of 16.7 h from patient admission to the start of surgery. Regarding transfer from the scene of accident, we found that the median time from injury to NTC admission was 5.6 h. In total, our results indicate that the full potential of the SENHR trauma system to provide surgery within the recommended 24 h to patients with traumatic cSCI was not utilized.

Several studies have demonstrated a clinically advantageous impact of early surgical decompression for cSCI (1–5). The STASCIS trial showed that spinal cord decompression within 24 h after injury increased the odds of at least a two-grade AIS improvement 6 months after injury 2.8 times as compared to patients who underwent late surgery. A later pooled analysis of individual patient data from four different data sets was concluded that surgery within 24 h was associated with improved sensorimotor recovery (23). This has led to the development of a practice guideline for the treatment of cSCI (6), which we have implemented at OUH. For the subset of patients suffering from CCS, the current literature indicates that the same treatment policy should also be provided for this specific condition. Although an RCT has not been conducted for CCS, there is some evidence suggesting that CCS should also be treated in a NICU with appropriate medical surveillance and interventions, alignment and stabilization of fractures, and surgical decompression to alleviate pressure on the affected spinal cord (22). However, it must be emphasized that we have only level-2 evidence for the treatment recommendations based on cohort studies since, as of yet, there are no evidence-based studies that establish the indications and timing of surgery for cSCI (24).

Based on the available literature and treatment recommendations, our department has decided to follow the established principles advocating early surgery in the treatment of cSCI. So far, there has been a knowledge gap about our adherence to the treatment recommendations. We have hypothesized that with the current Norwegian trauma system and the 24/7 transfer options to the NTC, starting surgery in less than 24 h after injury should be achievable. In planning the study, we realized that in the chain of management, there are two distinct time periods that must be looked at separately: (1) time from the scene of accident to NTC admission and (2) time from NTC admission to the start of surgery. To identify possible factors associated with time delays and thus to facilitate improvement of the service, we performed separate regression analyses of the two time periods.

### 4.1 Regarding time from injury to NTC admission

The NTC at OUH covers a large geographical region (111.019 square km), almost three times the size of Denmark’s land area (25), and where the largest cross-sectional distance is around 500 km. Therefore, we believe that the overall function of the transfer system within the region is highly efficient since the median time from injury to NTC admission was as short as 5.6 h (IQR 1.9–19.4 h). Within 12 and 24 h after injury, 67 and 79% of the patients, respectively, were admitted to the NTC. Not surprisingly, we found that for patients first triaged at local hospitals, there was a significant increased time from trauma to NTC admission (*p* < 0.001, 95% CI 1.5–2.1). In fact, the majority of patients were first triaged at LHs (62%), which may be an indication of a heterogeneous clinical presentation of the cSCI and a long distance to the NTC. Given clinical suspicion of all types of cSCI at the trauma scene, direct transfer to the NTC is implemented as standard operating procedure (SOP). This is in line with the recently updated guideline for the field triage of injured patients (26). One might argue that some of these patients may have multiple injuries in need of stabilization at an LH located closer to the injury scene before transfer to the trauma center. However, in the study, we found that absence of multiple injuries was a predictor for delayed transfer (*p* < 0.001, 95% CI 0.74–0.21), meaning that to a higher degree, patients with multiple injuries are transferred directly to the NTC. To some surprise, we found that patients with severe preinjury comorbidities (ASA ≥ 3) had increased time from injury to NTC admission (*p* = 0.015, 95% CI 0.08–0.69). We have no clear explanation for this result. For patients first triaged at LHs, we recommend that contact with the NTC must be established as soon as possible and that evaluation with MRI in most cases should be performed at the NTC in order to avoid diagnostic delay at the LH and to optimize the quality of MRI.

### 4.2 Regarding time from NTC admission to the start of surgery

In light of the short median time interval from trauma to NTC admission, we found a surprising median of 16.7 h from NTC admission to surgery onset. The only factors significantly associated with a time delay were increasing age and non-translational injury. We find this puzzling since potential obstacles for early surgery, like preinjury comorbidity, AIS grade, and multiple injuries, were not associated with a time delay. Therefore, it seems likely that improvement of the in-house logistics at the NTC will contribute substantially to reducing the door-to-surgery time.

In order to safely perform surgery on cSCI patients, several issues have to be addressed in the preoperative phase. At our institution, all trauma patients are treated according to the ATLS principles (27), with appropriate imaging, medical stabilization, MRI, and intubation when deemed necessary. Current neurosurgical SOP of cSCI is evaluation with both CT and MRI of the cervical spine and CT angiography of prevertebral vessels for patients with CS-fx. Of these, MRI is usually the most time-consuming; however, a full scan should be completed within an hour. At OUH, the CT is available in the ER, but for MRI, the patients must be transported to the radiological department. Further, the induction of general anesthesia and proper positioning of the patient on the operating table is time-consuming and often takes about 1 h. Thus, a conservative estimate of the minimum preoperative time consumption from door-to-knife in an optimal fast-track chain for cSCI without other major trauma needing intervention at our institution could be around 4 h (ER 45 min, CT 30 min, MRI 60 min, in-hospital transfer from ER to MRI to OR 45 min, preoperatively in OR 60 min).

It is well known that the availability of MRI could pose a challenge, especially after ordinary working hours. Further, the neurosurgical on-call team may be occupied with other operations, meaning that a cSCI patient needing surgery after ordinary working hours may have to wait for the first available OR. Altogether, in order to reduce in-house time consumption at the NTC before surgery in patients with cSCI, we suggest that MRI should be requested at first contact with the neurosurgeon on-call and that patients be transferred for MRI directly from the ER after initial assessment, necessary resuscitative treatment, and CT scanning to safely tolerate the procedure.

Clearly, if MRI is omitted and the cSCI patients are investigated only with CT, it would be possible to reduce the overall preparation time before surgery. Hence, to speed up the process, some clinicians may argue that CT is sufficient. However, in our view, important structural injuries, such as disc herniations, intraspinal hematoma, cerebrospinal fluid leaks, medullary edema, and medullary disruption, may be missed without MRI. Supporting this routine, research has shown that the addition of preoperative MRI significantly changes the surgical plan in adult patients with traumatic cSCI (28). Consequently, we will continue the routine of evaluating all cSCI patients with MRI before surgery.

Moreover, the neurosurgical team should schedule fixation and decompression of the cSCI for the same calendar day as the injury or as the first operation the following morning for patients admitted late in the evening or night. One might argue that for older patients with comorbidity, it is necessary to wait until the next daylight to reduce potential perioperative complications; however, in a recent study of 87 patients older than 70 years with cSCI, urgent surgery within 8 h after arrival at the NTC did not result in increased perioperative complications (29). Furthermore, the motor index score was significantly improved in the early surgery group. Thus, it seems that a fast-track chain for cSCI surgery is both safe and feasible for all age groups.

The major strength of our study is that it is population-based and uses data from a recent series of patients. None of the operations were performed at the LHs in this defined geographical region; therefore, all potential patients were included in the study. The main limitations are the retrospective collection of patient and radiology data to complete the already registered data in the prospective enrollment of the patients in the registry and that 27% of the patients with cSCI did not have surgery. Moreover, we have not assessed the degree of ongoing medullary compression on NTC admission. Furthermore, we cannot rule out that some patients with cSCI were not referred to the NTC.

## 5 Conclusion

Less than half of patients with cSCI were operated on within 24 h after injury. We support early surgery in line with current treatment recommendations. We have identified several possible ways to improve the management of cSCI patients in our institution: (1) patients with clinical suspicion of cSCI should be transported directly to the NTC from the scene of accident, (2) for patients initially triaged at LHs, the stay should be shortened and the MRI should be performed at the NTC, and (3) after arrival at the NTC, surgery should commence promptly after MRI and ideally on the same calendar day as arrival.

## Data availability statement

The raw data supporting the conclusions of this article will be made available by the authors, without undue reservation.

## Author contributions

MA: Conceptualization, Formal analysis, Writing – original draft, Writing – review & editing. JM: Writing – review & editing. PRø: Data curation, Formal analysis, Writing – review & editing. MS: Writing – review & editing. TG: Writing – review & editing. SR: Writing – review & editing. DB: Writing – review & editing. HØ: Writing – review & editing. PRy: Writing – review & editing. VS: Writing – review & editing. PN: Writing – review & editing. CG: Writing – review & editing. TB: Writing – review & editing. HL: Writing – review & editing. EH: Writing – review & editing, Conceptualization, Data curation, Project administration, Writing – original draft.
